# Construction and validation of a cognitive frailty risk prediction model for elderly patients with colorectal cancer

**DOI:** 10.3389/fnagi.2025.1692414

**Published:** 2025-12-01

**Authors:** Yu Wang, Li Wang, Yunhong Du, Xiaoye Ma, Lili Sun, Xiujie Zhang, Wenli Rong, Jianwei Li, Yao Shi, Wei Liu, Danqi Xie, Lili Peng, Ouying Chen

**Affiliations:** 1 School of Nursing, Hunan University of Chinese Medicine, Changsha, China; 2 Nursing Department, Qingdao Traditional Chinese Medicine Hospital (Qingdao Hiser Hospital Affiliated of Qingdao University), Qingdao, China; 3 Nursing Department, The First Affiliated Hospital of Dalian Medical University, Dalian, China; 4 Cardiovascular Surgery Intensive Care Unit, The First Affiliated Hospital of Naval Medical University, Shanghai, China; 5 The First Clinical Medical College, Shandong University of Traditional Chinese Medicine, Jinan, China

**Keywords:** elderly patients, colorectal cancer, cognitive frailty, risk prediction model, nomogram

## Abstract

**Background:**

Elderly patients with colorectal cancer (CRC) are a high-risk population for cognitive frailty (CF). This study aims to develop and validate a risk prediction model for CF in this specific patient group, so as to facilitate early identification and intervention.

**Methods:**

This study collected cross-sectional data from 528 elderly patients with CRC who were treated in multiple Grade A Class 3 hospitals in Shandong Province from July 2024 to July 2025. A total of 22 indicators were included. Logistic regression was employed to identify factors associated with CF in elderly patients with colorectal cancer, and R software (version 4.4.3) was used to develop a risk prediction model. The predictive performance and clinical utility of the model were evaluated using metrics including the area under the receiver operating characteristic curve (AUC), calibration curve, and decision curve analysis (DCA).

**Results:**

The regression analysis showed that age, chemotherapy history, tumor stage, whether engaging in intellectual activities, social support level, and educational level were independent risk factors for CF in elderly patients with CRC (*p* < 0.05). The AUC of the modeling group and the validation group was 0.819 and 0.802, respectively; the Hosmer-Lemeshow test results indicated good model fit; the consistency between the actual values and the predicted values of the calibration curve was also high.

**Conclusion:**

The risk of CF in elderly patients with colorectal cancer is relatively high, and it is related to factors such as age, chemotherapy history, tumor stage, whether engaging in intellectual activities, social support level, and educational level. The risk prediction model for CF in elderly patients with CRC developed in this study exhibits good predictive performance in both internal and external validations. The prediction model constructed in this study can provide a reference for healthcare providers to early identify high-risk individuals and implement targeted intervention measures.

## 1 Introduction

With the acceleration of global population aging, healthy aging has become a core issue in the field of geriatrics. With the deepening of research on the relationship between physical frailty and cognitive dysfunction (Han et al., 2021; Zeng, 2021; Feng et al., 2017; Cui et al., 2023; Yu et al., 2018; Simó et al., 2017; Margioti et al., 2020), cognitive frailty (CF), as a key phenotype of geriatric syndromes, has gradually become the focus of clinical attention. CF is one of the subtypes of generalized frailty, which refers to the pathophysiological process of CF and frailty. It is characterized by the coexistence of physical frailty and mild cognitive impairment, and excludes Alzheimer’s disease or other types of dementia (Kelaiditi et al., 2013). Its occurrence not only affects patients’ understanding and compliance with treatment regimens, but also significantly increases the risk of adverse outcomes such as falls (Ma et al., 2021; Rivan et al., 2021), disability (Rivan et al., 2021; Tsutsumimoto et al., 2020), depression (Yoshiura et al., 2022), and prolonged hospitalization (Yu et al., 2018).

Colorectal cancer (CRC) is the third most common cancer and the second leading cause of cancer-related death worldwide., with a high incidence in the elderly (Bray et al., 2024; Zhang et al., 2023). The Cancer Treatment and Survivors Statistics show (Miller et al., 2022) that nearly four out of five CRC patients are over 60 years of age. The incidence of physical frailty in this population is as high as 60.5% due to factors such as decreased digestive and absorption function, chronic inflammatory response and immune function suppression, which is significantly higher than that of other digestive system tumors (Zhang H. B. et al., 2022; Michaud Maturana et al., 2021). Compared with other cancers, CRC cells can destroy the homeostasis of intestinal flora during the occurrence and development, and intestinal flora indirectly affects the cognitive function of the brain through neural, immune, endocrine and metabolic pathways through the brain-gut axis (Zhang Y. L. et al., 2022), leading to a significant increase in the risk of cognitive dysfunction in this population. Therefore, the dual threat of physical frailty and cognitive dysfunction makes elderly CRC patients a high-risk group for CF.

Although CF is regarded as a reversible stage of neurodegenerative diseases and a golden window for early intervention (Kelaiditi et al., 2013; Panza et al., 2017), there are still deficiencies in the assessment and management of CF in elderly patients with CRC in current clinical practice. The existing studies mostly focus on single disease or single dimension assessment, and lack systematic exploration of the synergistic mechanism of cognitive function and frailty. At the same time, the lack of standardized risk prediction tools suitable for CRC specialist scenarios makes it difficult to achieve early identification and precise intervention of high-risk patients.

By integrating multi-dimensional influencing factors, risk prediction models can scientifically estimate the probability of occurrence of a disease or outcome and provide quantitative basis for clinical decision-making (Zhang et al., 2015). On the basis of previous studies, this study will systematically screen the key predictive variables and construct a more accurate risk prediction model for CF in elderly patients with CRC based on the correlation between disease characteristics and CF, so as to provide an assessment tool for clinical workers to early identify high-risk groups and a theoretical basis for further implementation of targeted nursing interventions.

## 2 Materials and methods

### 2.1 Study subjects

Using the convenient sampling method, 367 elderly patients with CRC treated in two Grade A Class 3 hospitals in Shandong Province from July 2024 to March 2025 were selected as the modeling group, and 161 elderly patients with CRC treated in three secondary first-class hospitals in Shandong Province from April 2025 to July 2025 were selected as the validation group.

- Inclusion criteria: CRC patients who met the diagnostic criteria of “Chinese Guidelines for Diagnosis and Treatment of CRC (2023 Edition)” (NHC, M. o. and OBCMA, 2023); age ≥ 60 years old; Communication skills barrierless, able to clearly express their wishes; voluntarily participate in this study and sign the informed consent form, and voluntarily accept the relevant investigation of this study.
- Exclusion criteria: Patients with severe primary diseases of liver, kidney, hematopoietic system, endocrine system and other serious organ failure; patients with psychiatric history, personality disorder history, cognitive impairment, and organic brain diseases; patients taking medication for mental disorders; severe vision and hearing impairment; and patients who had participated in other trials or were participating in other trials within 3 months before obtaining informed consent.
- Exclusion criteria: poor questionnaire completion quality (data missing rate >10%); poor compliance and distorted results due to emotion or other reasons; patients voluntarily withdraw from the study or sudden deterioration of the condition.

This study used the calculation method of sample size formula in epidemiological cross-sectional studies (Zheng and He, 2020), $n=\frac{Z_{1-\alpha /2}^{2}\cdot P\cdot (1-P)}{d^{2}}$ . According to the preliminary investigation, the risk of CF in this population is 30%. Under the assumption of CI 95% and d 5%, we used the method of two-sided test. The equation Z_1-α/2_ is 1.96; *p* = 0.30; *d* = 0.05. At the same time, 10% of the sample missing rate was considered, and the sample size of the modeling group was at least 359 cases. *δ* is the allowable error, which was controlled at 5% in this study. The sample size of the validation group of risk prediction model is generally 1/4–1/2 of the sample size of the modeling group (Jiang, 2023), and the invalid questionnaires are 10%, so the sample size of the validation group should be at least 102 cases. This study has received ethical approval (2024HC09LS008). All patients voluntarily participated in this study and signed informed consent.

Based on the study population and the popularity of assessment tools, this study selected the currently widely used Fried Frailty Phenotype (FP) criteria and Montreal Cognitive Assessment (MoCA) scale, and combined them with the Clinical Dementia Rating (CDR) scale to conduct CF assessment, so as to ensure the accuracy and feasibility of the assessment. According to the diagnostic criteria of CF (Kelaiditi et al., 2013) formulated by the International Society of Nutrition and Aging and the International Society of Geriatrics, the following criteria were made for CF: patients or caregivers complained of CF, CDR rating scale score = 0.5; FP score > 3; MoCA score < 26; Alzheimer’s disease and other types of dementia were excluded. If all the above conditions were met, CF was diagnosed.

### 2.2 Research tools

#### 2.2.1 Self-made questionnaire

By consulting relevant literature in domestic and foreign databases, the influencing factors of CF in patients with CRC were screened. Combined with the results of expert meetings and pre-survey, and guided by the theory of health ecology, the General Information Questionnaire was finally formed. The specific contents included. Personal trait layer: The data included gender, age, body mass index (BMI), nutritional status, history of surgery, radiotherapy, chemotherapy, tumor stage, metastasis, Charlson comorbidity index (CCI), carcinoembryonic antigen (CEA), carbohydrate antigen 19–9 (CA19-9) and serum albumin level; Behavior and psychological layer: including smoking history (using a continuous or cumulative smoking duration of 6 months as the cutoff), drinking history (using a consumption of >100 mL of baijiu per occasion with continuous drinking for 1 year as the cutoff), whether to carry out intellectual activities (intellectual activities encompassed cognitively stimulating pursuits such as internet use, newspaper reading, calligraphy, painting, musical instrument playing, chess, and mahjong), depression, sleep duration; Interpersonal network layer: marital status, social support level; living and working conditions: education level, family per capita monthly income.

#### 2.2.2 Variables assessment tool

- Patient-Generated Subjective Global Assessment (PG-SGA): It is a nutritional status assessment form specially designed for cancer patients, which uses a comprehensive method to evaluate their nutritional status (Hsieh et al., 2016). The instrument includes both patient self-assessment and provider assessment with a total score ranging from 0 to 35. Only the patient self-assessment scale part was used in this study, with higher scores indicating worse nutritional status, divided into A = well nourished (0 to 3 points), B = suspected or moderate malnutrition (4 to 8 points), and C = severe malnutrition (>8 points). The scale showed that the sensitivity was 87.6%, the specificity was 83.1%, and the Cronbach’s α coefficient was 0.712 (Hua et al., 2021).
- Geriatric Depression Scale-15 (GDS-15): contains 15 items (Sheikh and Yesavage, 1985), and the patient’s answer to each item is “yes” or “no,” and the answer to “no” in the 1st, 5th, 7th and 11th items is 1 point, and the answer to “yes” in the other items is 1 point. Total scores range from 0 to 15, with scores ≥5 indicating depression. The Cronbach’s α coefficient of the Chinese version was 0.793 (Tang, 2013).
- Social Support Rating Scale (SSRS): This scale has 10 items and 3 dimensions, namely objective support, social support utilization and subjective support (Xiao, 1994). The total score ranges from 12 to 66 points, the score of objective support dimension was 4–16, the score of subjective support dimension was 5–38, and the score of support utilization dimension was 3–12. The higher the score, the more support the individual received. According to the score, it can be divided into three levels: the low level is 12–22 points, the middle level is 23–44 points, and the high level is 45–66 points. The scale had good reliability and validity, with Cronbach’s α coefficient of 0.91 (Xiao, 1994).
- CCI: In this study, CCI (Charlson et al., 1987) was used for comorbidity assessment. Comorbidity score: 1 point disease: Hypertension, coronary atherosclerotic heart disease, congestive heart failure, chronic lung disease, peptic ulcer, chronic liver disease or diabetes mellitus (without complications); Grade 2 disease: hemiplegia, moderate or severe kidney disease, diabetes mellitus (with complications), malignant tumors including leukemia or malignant lymphoma; Grade 3 disease: moderate or severe liver disease; Grade 6 diseases: malignant tumor with metastasis, AIDS; Age score: those over 50 years old increased by 1 point every 10 years. The CCI score was calculated by adding the comorbidity score and age score, and was divided into three grades: low (2–3 points), moderate (4–5 points) and severe (≥6 points). The scale has good reliability and validity, and Cronbach’s α coefficient is 0.864 (Jiang, 2023).

#### 2.2.3 CF evaluation tools

- FP: developed by Fried et al. (2001). The scale contains 5 items: weight loss, gait speed reduction, grip strength reduction, fatigue, and physical activity reduction. One point is assigned for each item met, with a total score ranging from 0 to 5. A score of 0 indicates no frailty; a score of 1–2 indicates pre-frailty; and a score of 3–5 indicates physical frailty. The Cronbach’s α coefficient was 0.89 (Wang et al., 2016).
- MoCA: This scale was developed by Canadian scholar Nasreddine et al. (2005), and the final version was determined in 2004. MoCA scale has a variety of different language versions and is widely used in the world. The scale covers eight cognitive domains, including visuospatial function, naming, attention, repetitive sentences, fluency, abstract ability, delayed recall, and orientation. The total score is the sum of the scores of each cognitive domain, and the score is 0–30 points. If the education is less than 12 years, the total score is added by 1 point. A score ≥26 was defined as no cognitive impairment. The Cronbach’s α coefficient was 0.933 (Xia et al., 2021).
- CDR scale: the evaluation content includes 6 aspects: judgment and problem-solving ability, orientation, memory, social affairs, housework and hobbies, and personal self-care ability (Hughes et al., 1982). The evaluation procedure was carried out by specially trained clinical medical staff, who interviewed patients and their caregivers, and scored CDR based on the information and clinical changes obtained from the interviews, with Cronbach’s α coefficient of 0.873 (Yang et al., 2018).

### 2.3 Collection of data and quality control

The investigators were trained before the survey, including survey skills, scoring methods of survey tools and precautions, etc. The instructions used were unified and standardized. Data collection simulation exercises and training examinations were conducted for each investigator to ensure the accuracy and consistency of the data collected by each investigator. This study collected data using a combination of face-to-face questionnaire surveys and data extraction from the electronic medical record system. Research investigators distributed questionnaires on-site, explained the filling instructions, and addressed questions raised by the participants. The questionnaires were collected promptly on-site, and their content was verified. Questionnaires with excessively short completion time, obvious response regularity, filling errors or irregularities, and missing items were all excluded. If missing items were identified during on-site inspection, the investigators communicated with the patients promptly to complete the missing information to ensure the accuracy and completeness of the questionnaires. Questionnaires with no issues were filed for documentation. The same equipment and method were used to measure the parameters of the patients. The following kinds of measuring equipment are mainly used, namely, soft ruler, scale, electronic stopwatch (Li Ning YQDT019) and electronic grip strength meter (Xiangshan EH101). During the assessment of gait speed, the patient could walk twice at a normal pace on a flat surface of 4.57 meters, and the average gait speed was taken. To evaluate the grip strength, the patient should take a standing position with the upper limbs sagging naturally and grip the dynamometer twice to obtain the average grip strength. The electronic medical record was reviewed, and the general information, comorbidities, and laboratory indicators of the subjects were collected. The BMI of the subjects was calculated by the researchers. The principal investigator of the study organizes the data of patients who have completed the entire survey process. If there are abnormalities or missing information in the data, the PI contacts the investigator who collected the patient’s data, reviews the patient’s electronic medical record information, and supplements and verifies the missing or abnormal parts of the content. Double entry and reverse check were used to input the collected research data into Excel software, and the Vlookup function and the data duplication function in Excel software were used to double check the data.

### 2.4 Statistical methods

SPSS 27.0 and R4.4.3 software were used for statistical analysis. The distribution characteristics of continuous variables satisfying normality were described by mean ± standard deviation ( $\bar{x}\pm s$ ), and the independent sample t test was used for comparison between groups. The distribution characteristics of continuous variables that did not meet the normality were described by median and interquartile range (IQR), and the non-parametric rank sum test was used for comparison between groups. The distribution characteristics of categorical variables were described by frequency and percentage, and the chi-square test or Fisher exact probability method was used for comparison between groups. In order to reduce multicollinearity and overfitting, LASSO regression was used for preliminary screening of predictors. Variables with regression coefficient not 0 were analyzed by univariate analysis, and then the significant influencing factors of univariate analysis (*p* < 0.05) were analyzed by multivariate analysis. The variance inflation factor (VIF) were used to check for the presence of multicollinearity among independent variables. The final screening predictors established a risk prediction model for CF in elderly patients with CRC, and the test level was set as α = 0.05. The Bootstrap repeated sampling method was used for internal verification of the modeling group, and the data of the validation group were collected for external verification. The area under the receiver operating characteristic curve (AUC) was used to evaluate the discrimination of the model. The Hosmer-Lemeshow (H-L) test, calibration curve and Brier score were used to evaluate the calibration. Decision curve analysis (DCA) was used to evaluate the clinical effectiveness of the model. A nomogram was drawn to visualize the model.

## 3 Results

### 3.1 Current status of CF in elderly patients with CRC

**Figure 1:**
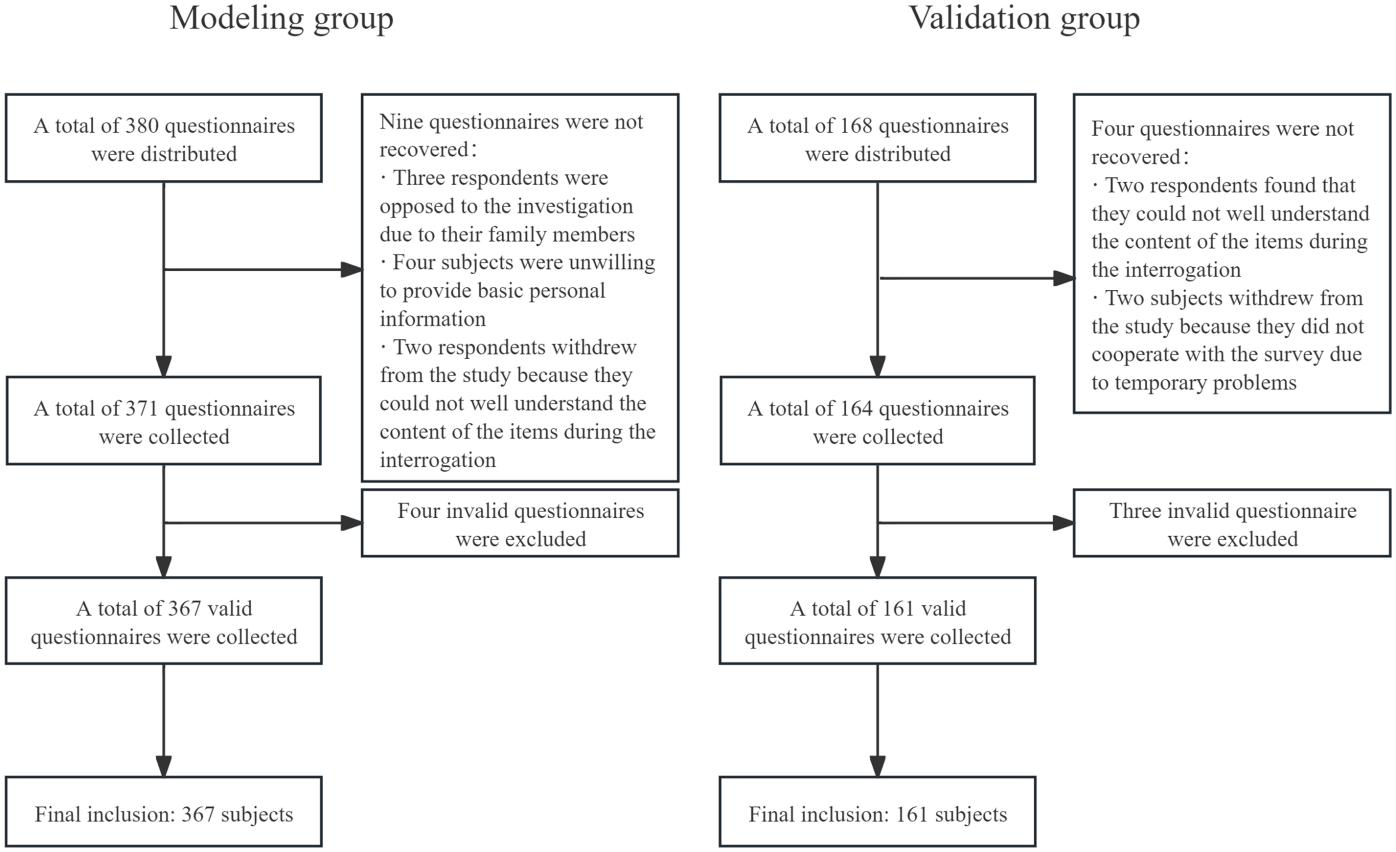
Flowchart of the inclusion process for the modeling group and the validation group study subjects.

During data collection in the modeling group, 380 questionnaires were distributed, 371 questionnaires were returned, and 367 were valid, with an effective recovery rate of 96.6% (see Figure 1 for details). Among 367 elderly patients with CRC in the modeling group, there were 186 individuals with a CDR score of 0.5, 163 individuals with an FP score of ≥3, and 155 individuals with a MoCA score of <26. There were 131 patients in the CF group and 236 patients in the non-CF group, with an actual incidence of CF of 35.7%.

**Figure 2:**
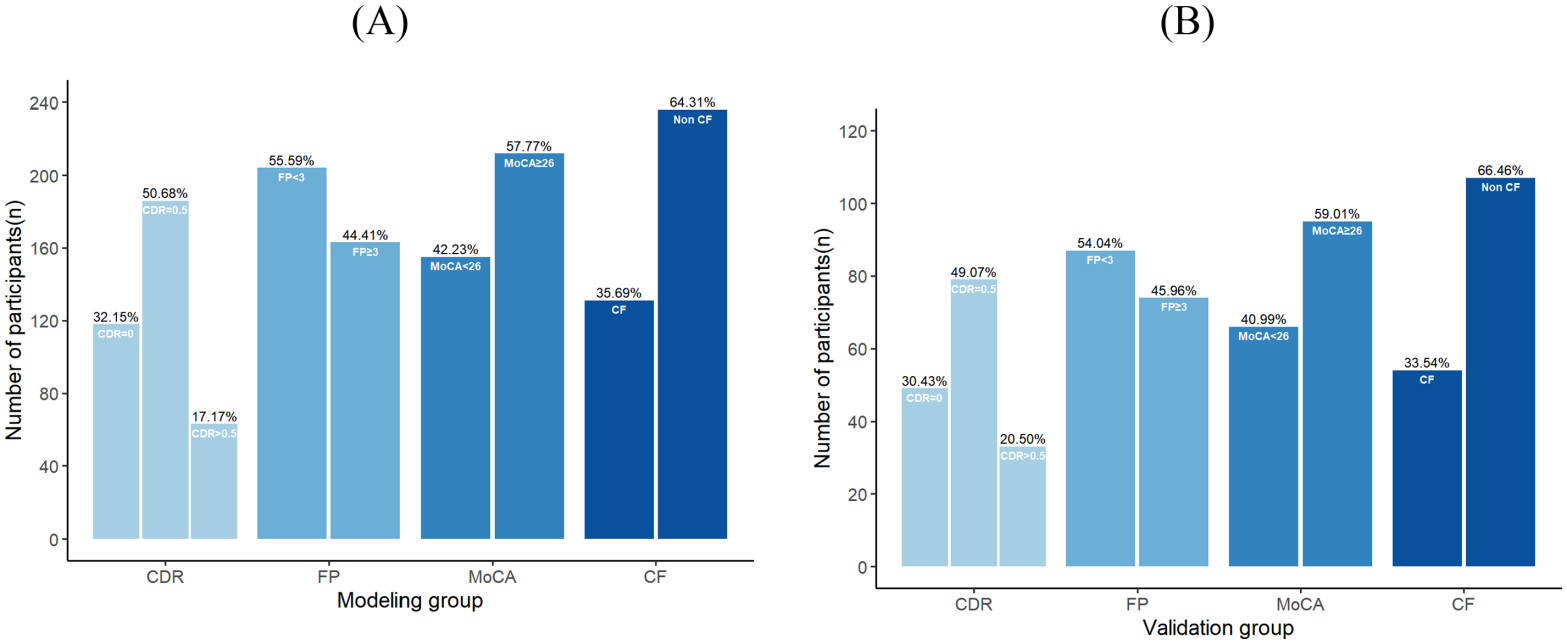
**(A)** The detailed distribution of CDR, FP, and MoCA scores in modeling group. **(B)** The detailed distribution of CDR, FP, and MoCA scores in validation group.

At the time of data collection for the validation group, a total of 168 questionnaires were sent out and 164 were returned, with 161 valid questionnaires, and the effective recovery rate of the questionnaire was 98.1%. Among the 161 elderly patients with CRC in the validation group, the CDR score was 0.5 points for 79 individuals, the FP score was ≥3 points for 74 individuals, and the MoCA score was <26 points for 66 individuals. Fifty-four were in the CF group and 107 were in the non-CF group, and the actual incidence of CF was 33.5%. The detailed distribution of CDR, FP, and MoCA scores is shown in Figure 2.

### 3.2 General data of elderly patients with CRC

A total of 378 participants were included in the modeling group, with an age range of 60–87 years. Among them, 163 cases (44.4%) were at tumor stage I–II, 121 cases (33.0%) at stage III, and 83 cases (22.6%) at stage IV; 283 cases (74.9%) had a chemotherapy history. The validation group included 161 participants, with an age range of 60–85 years. Among them, 78 cases (48.4%) were at tumor stage I–II, 50 cases (31.1%) at stage III, and 33 cases (20.5%) at stage IV; 121 cases (75.2%) had a chemotherapy history.

There was no statistically significant difference in general demographic data and disease characteristics between the two groups (*p* > 0.05), indicating that their baseline data were basically consistent. Specific information is shown in Table 1.

**Table 1:** Distribution of baseline characteristics in the modeling and testing groups [*n*(%)/M(*P*_25_, P_75_)].

Variables	Classification	Modeling group (*n* = 367)	Validation group (*n* = 161)	χ^2^/*Z* value	*p* value
Educational level, *n* (%)				1.212^a^	0.545
	College or above	47 (12.8)	23 (14.3)		
	Middle School/High School	146 (39.8)	70 (43.5)		
	Primary school and below	174 (47.4)	68 (42.2)		
Income, *n* (%)				1.077^a^	0.584
	>5,000 yuan	58 (15.8)	28 (17.4)		
	2,500–5,000 yuan	171 (46.6)	80 (49.7)		
	<2,500 yuan	138 (37.6)	53 (32.9)		
Marital status, *n* (%)				0.783^a^	0.376
	Married	255 (69.5)	118 (73.3)		
	Unmarried/Divorced/Widowed	112 (30.5)	43 (26.7)		
Level of social support, *n* (%)				0.447^a^	0.800
	High level	71 (19.3)	34 (21.1)		
	Moderate level	158 (43.1)	71 (44.1)		
	Low level	138 (37.6)	56 (34.8)		
Whether to carry out intellectual activities, *n* (%)				0.395^a^	0.530
	Yes	192 (52.3)	89 (55.3)		
	No	175 (47.7)	72 (44.7)		
Depression, *n* (%)				0.764^a^	0.382
	No	234 (63.8)	109 (67.7)		
	Yes	133 (36.2)	52 (32.3)		
Sleep, *n* (%)				0.944^a^	0.331
	≥6 h	223 (60.8)	105 (65.2)		
	<6 h	144 (39.2)	56 (34.8)		
Smoking history, *n* (%)				1.194^a^	0.274
	No	266 (72.5)	124 (77.0)		
	Yes	101 (27.5)	37 (23.0)		
Drinking history, *n* (%)				0.469^a^	0.493
	No	288 (78.5)	122 (75.8)		
	Yes	79 (21.5)	39 (24.2)		
Gender, *n* (%)				0.469^a^	0.494
	Man	214 (58.3)	99 (61.5)		
	Woman	153 (41.7)	62 (38.5)		
BMI, *n* (%)				0.677^a^	0.713
	≥24	79 (21.5)	37 (23.0)		
	18.5–23.999	201 (54.8)	91 (56.5)		
	<18.5	87 (23.7)	33 (20.5)		
Nutritional status, *n* (%)				0.467^a^	0.792
	Good nutrition	121 (33.0)	58 (36.0)		
	Mild to moderate malnutrition	151 (41.1)	63 (39.1)		
	Severe malnutrition	95 (25.9)	40 (24.8)		
Operation history, *n* (%)				0.572^a^	0.449
	No	78 (21.3)	39 (24.2)		
	Yes	289 (78.7)	122 (75.8)		
Radiotherapy history, *n* (%)				0.383^a^	0.536
	No	298 (81.2)	127 (78.9)		
	Yes	69 (18.8)	34 (21.1)		
Chemotherapy history, *n* (%)				0.238^a^	0.625
	No	84 (22.9)	40 (24.8)		
	Yes	283 (77.1)	121 (75.2)		
Tumor stage, *n* (%)				0.734^a^	0.392
	I–II stage	163 (44.4)	78 (48.4)		
	III–IV stage	204 (55.6)	83 (51.6)		
Migration, *n* (%)				0.293^a^	0.588
	No	284 (77.4)	128 (79.5)		
	Yes	83 (22.6)	33 (20.5)		
CCI, *n* (%)				0.466^a^	0.792
	2–3 points for low comorbidities	142 (38.7)	63 (39.1)		
	4 to 5 points for comorbidities	131 (35.7)	61 (37.9)		
	High comorbidity score ≥ 6	94 (25.6)	37 (23.0)		
Age, M [IQR]		69 (65, 74)	69 (65, 73)	−1.479^b^	0.139
ALB, M [IQR]		40.9 (34.6, 44.5)	41.4 (36.5, 46.1)	−1.345^b^	0.179
CEA, M [IQR]		4.7 (3.4, 20.2)	4.6 (3.1, 17.5)	−1.187^b^	0.235
CA199, M [IQR]		23.08 (12.07, 35.14)	18.69 (8.87, 35.83)	−2.331^b^	0.020

a χ^2^ value.

b *z* value.

M [IQR], median [interquartile range]; BMI, Body Mass Index; CCI, Charlson Comorbidity Index; ALB, Albumin; CEA, Carcinoembryonic Antigen; CA199, carbohydrate antigen 19-9.

### 3.3 Influencing factors of CF in elderly patients with CRC

#### 3.3.1 LASSO regression

**Figure 3:**
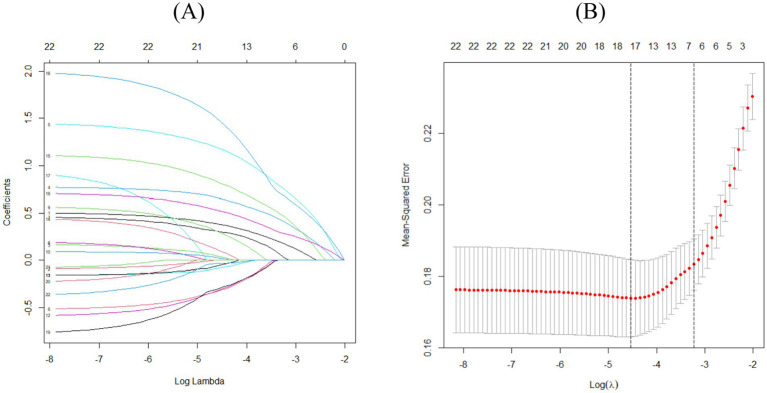
**(A)** Distribution plots of LASSO coefficients for each candidate variable. Each curve represents a different variable, and the ordinate on the left is the coefficient; when the coefficient is 0, it means that it is not included in the model. LASSO: Least Absolute Shrinkage and Selection Operator. **(B)** Plot of logarithm Log (λ) and partial likelihood deviation of adjustment parameter based on LASSO cross-validation. The number of key characteristic variables corresponding to different Log (λ) values is shown in the upper part of the figure. The dashed line on the left is the Log (λ) value corresponding to the minimum deviation, and the dashed line on the right is the Log (λ) value that is one standard error away from the minimum deviation.

In this study, LASSO regression was used to reduce the dimension of the collected 22 clinical characteristics to screen out the unimportant factors. To avoid overfitting, the coefficients of the independent variables are gradually compressed as the penalty coefficient *λ* changes, until the coefficients of some variables are compressed to zero. Figure 3 shows the LASSO coefficient distribution of each candidate variable in the screening process, and the final variable screening results are shown in Figure 3. According to the target variable, the predictors included in the model when λ = 0.03956771 were selected, and there were seven predictors included in the model, which were the individual trait level factors (age, chemotherapy history, tumor stage). Behavioral and psychological factors (whether to engage in intellectual activities, sleep duration), interpersonal network factors (social support level), living and working conditions (education level).

#### 3.3.2 Single factor analysis

Taking the occurrence of CF in elderly patients with CRC as the dependent variable, the seven predictors screened by Lasso regression were used as independent variables to conduct univariate analysis on the modeling group. Details as shown in Table 2. The results showed that there were significant differences in six variables including education level, intellectual activities, chemotherapy history, tumor stage, social support level and age (*p* < 0.05).

**Table 2:** Single factor analysis of the risk of CF in elderly patients with CRC.

Variables	Level	CF group (131)	Non CF group (236)	χ^2^ value	*P* value
Educational level, *n* (%)				12.21	0.002
	College or above	9 (6.9)	38 (16.1)		
	Middle school/high school	45 (34.4)	101 (42.8)		
	Primary school and below	77 (58.8)	97 (41.1)		
Level of social support, *n* (%)				19.097	<0.001
	High level	13 (9.9)	58 (24.6)		
	Moderate level	51 (38.9)	107 (45.3)		
	Low level	67 (51.1)	71 (30.1)		
Whether to carry out intellectual activities, *n* (%)				23.418	<0.001
	Yes	46 (35.1)	146 (61.9)		
	No	85 (64.9)	90 (38.1)		
Chemotherapy history, *n* (%)				14.091	<0.001
	No	15 (11.5)	69 (29.2)		
	Yes	116 (88.5)	167 (70.8)		
Tumor stage, *n* (%)				26.858	<0.001
	I–II stage	34 (26.0)	129 (54.7)		
	III–IV stage	97 (74.0)	107 (45.3)		
Age, *n* (%)				28.226	<0.001
	60–64 years old	16 (12.2)	60 (25.4)		
	65–69 years old	36 (27.5)	84 (35.6)		
	70–74 years old	30 (22.9)	59 (25.0)		
	75–79 years old	35 (26.7)	27 (11.4)		
	≥80 years old	14 (10.7)	6 (2.5)		
Duration of sleep, *n* (%)				2.162	0.141
	≥6 h	73 (55.7)	150 (63.6)		
	<6 h	58 (44.3)	86 (36.4)		

#### 3.3.3 Multiple factor analysis

Multivariable variables were analyzed by setting dummy variables. See Table 3 for the assignment of specific variables. Based on the AIC criterion, the variables were screened by the forward and backward method, and finally 6 independent influencing factors of CF in elderly patients with CRC were obtained (*p* < 0.05): education level, social support level, whether to carry out intellectual activities, chemotherapy history, tumor stage, and age (see Table 4 for details).

**Table 3:** Independent variable assignment.

Variables	Types of variables	Assignment of value
Chemotherapy history	Categorical variables	0 = No; 1 = Yes
Whether to carry out intellectual activities	Categorical variables	1 = Yes; 2 = No
Tumor stage	Categorical variables	1 = I–II stage; 2 = III–IV stage
Level of social support	Categorical variables	1 = high level; 2 = moderate level; 3 = low level
Educational level	Categorical variables	1 = college or above; 2 = middle school/high school; 3 = primary school and below
Age	Categorical variables	1 = 60–64; 2 = 65–69; 3 = 70–74; 4 = 75–79; 3 = ≥80

**Table 4:** Logistic regression analysis of the risk of CF in elderly patients with CRC.

Variables	Classification	β value	SE	Wald	*P* value	OR value	95% CI
Educational level	College or above			5.341	0.069		
	Middle school/high school	0.768	0.482	2.535	0.111	2.155	0.838–5.545
	Primary school and below	1.068	0.472	5.115	0.024	2.910	1.153–7.343
Level of social support	High level			15.214	<0.001		
	Moderate level	0.605	0.401	2.276	0.131	1.831	0.834–4.020
	Low level	1.416	0.402	12.424	<0.001	4.121	1.875–9.058
Whether to carry out intellectual activities		1.313	0.279	22.136	<0.001	3.718	2.151–6.425
Chemotherapy history		1.062	0.348	9.331	0.002	2.892	1.463–5.715
Tumor stage		1.151	0.275	17.561	<0.001	3.160	1.845–5.412
Age	60–64 years old			19.117	<0.001		
	65–69 years old	0.492	0.387	1.62	0.203	1.636	0.767–3.489
	70–74 years old	0.572	0.41	1.945	0.163	1.772	0.793–3.961
	75–79 years old	1.64	0.443	13.709	<0.001	5.157	2.164–12.288
	≥80 years old	1.856	0.671	7.659	0.006	6.400	1.719–23.829
Constant		−7.655	0.964	63.062	<0.001	0.005648	

OR, Odds Ratio; 95% CI, 95% Confidence Interval.

#### 3.3.4 Multicollinearity diagnosis

Multicollinearity of the above six variables was tested using VIF. The results showed that the tolerances of educational level, social support level, whether intellectual activities are performed, chemotherapy history, tumor stage, and age are all greater than 0.1, and the VIF values are all less than 5. This indicates no multicollinearity among the covariates, and all can be included in the prediction model (see Table 5).

**Table 5:** The collinearity diagnosis of predictors.

Variables	Allowance	VIF
Educational level	0.940	1.064
Level of social support	0.988	1.013
Whether to carry out intellectual activities	0.942	1.062
Chemotherapy history	0.981	1.020
Tumor stage	0.945	1.058
Age	0.947	1.056

VIF, Variance inflation factor.

### 3.4 To establish a prediction model for CF in elderly patients with CRC

The regression equation of CF risk prediction model for elderly patients with CRC was fitted to complete the model construction: Logit (p) = −7.655 + (1.068 × [assignment of primary school and below]) + (1.416 × [assignment of low level of social support]) + (1.313 × [intellectual activity]) + (1.062 × [chemotherapy history]) + (1.151 × [tumor stage]) + (1.640 × [age 75–79 years old]) + (1.856 × [age ≥80 years old]).

### 3.5 To evaluate the performance of CF prediction model in elderly patients with CRC

**Figure 4:**
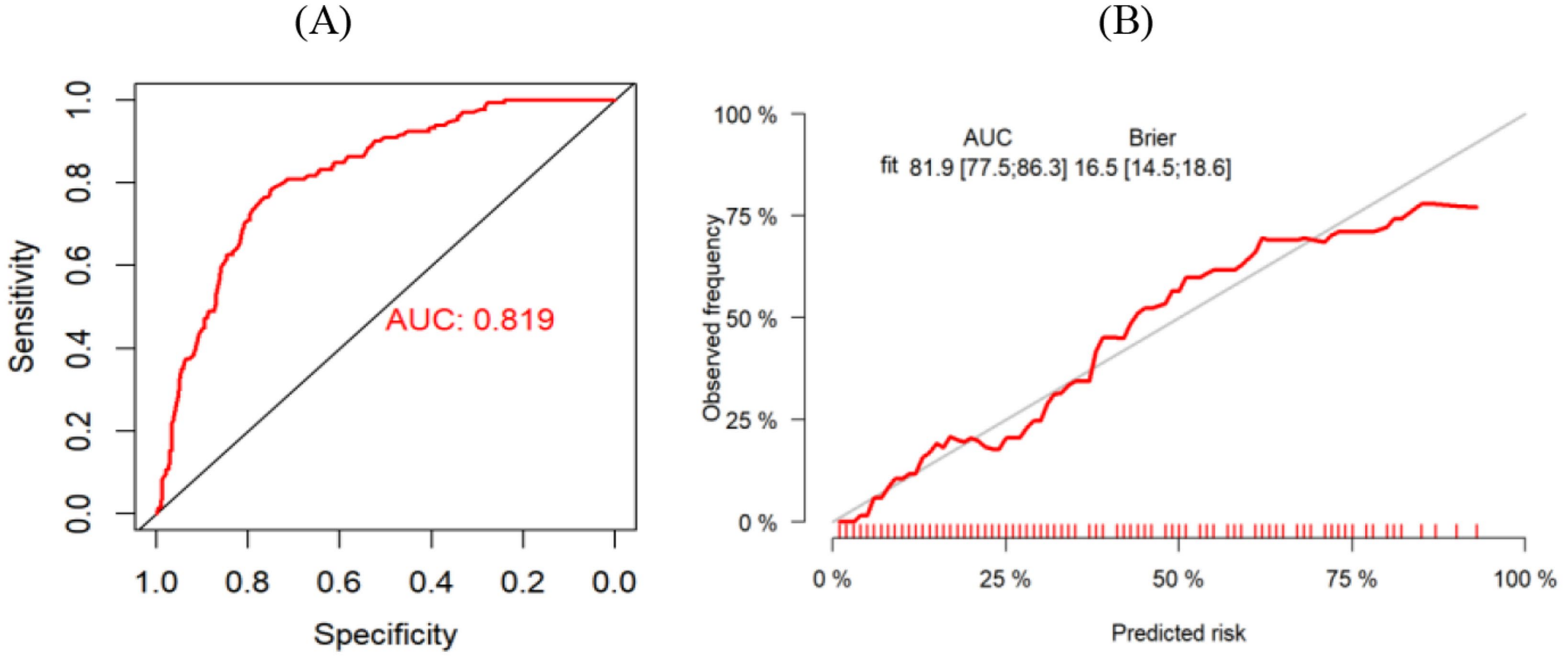
**(A)** ROC curve for risk prediction model for modeling group. **(B)** Calibration curves of the modeling group.

**Figure 5:**
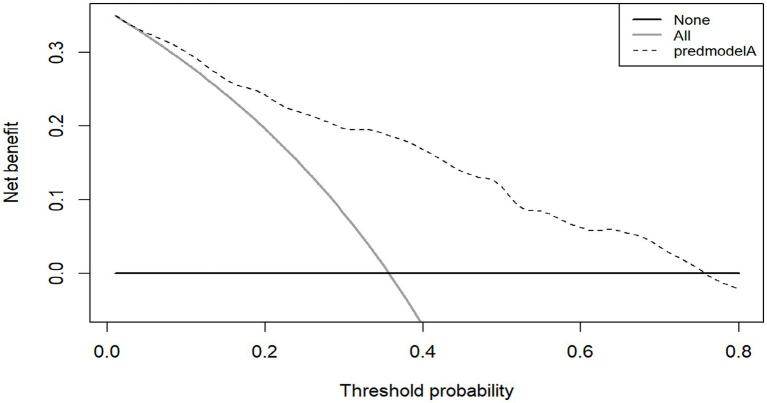
DCA of the modeling group.

The Bootstrap method was used to repeat sampling 1,000 times for internal validation of the data of the modeling group, and the AUC value was 0.819 (95% CI, 0.775–0.863) (Figure 4), and the Brier score was 0.165 (95% CI, 0.145–0.186). The results of the calibration curve showed that the probability of CF predicted by the model was in high agreement with the actual probability of CF (Figure 4), and the prediction was more accurate. H-L test χ^2^ value = 8.149, *p* = 0.49, indicating that there was no statistically significant difference between the predicted risk and the actual risk. In addition, the clinical validity of the model was assessed with the use of a decision curve (Figure 5), which yielded a net benefit of making clinical decisions that was greater than “no intervention” or “all intervention” options across probability thresholds of 0.05 to 0.75.

**Figure 6:**
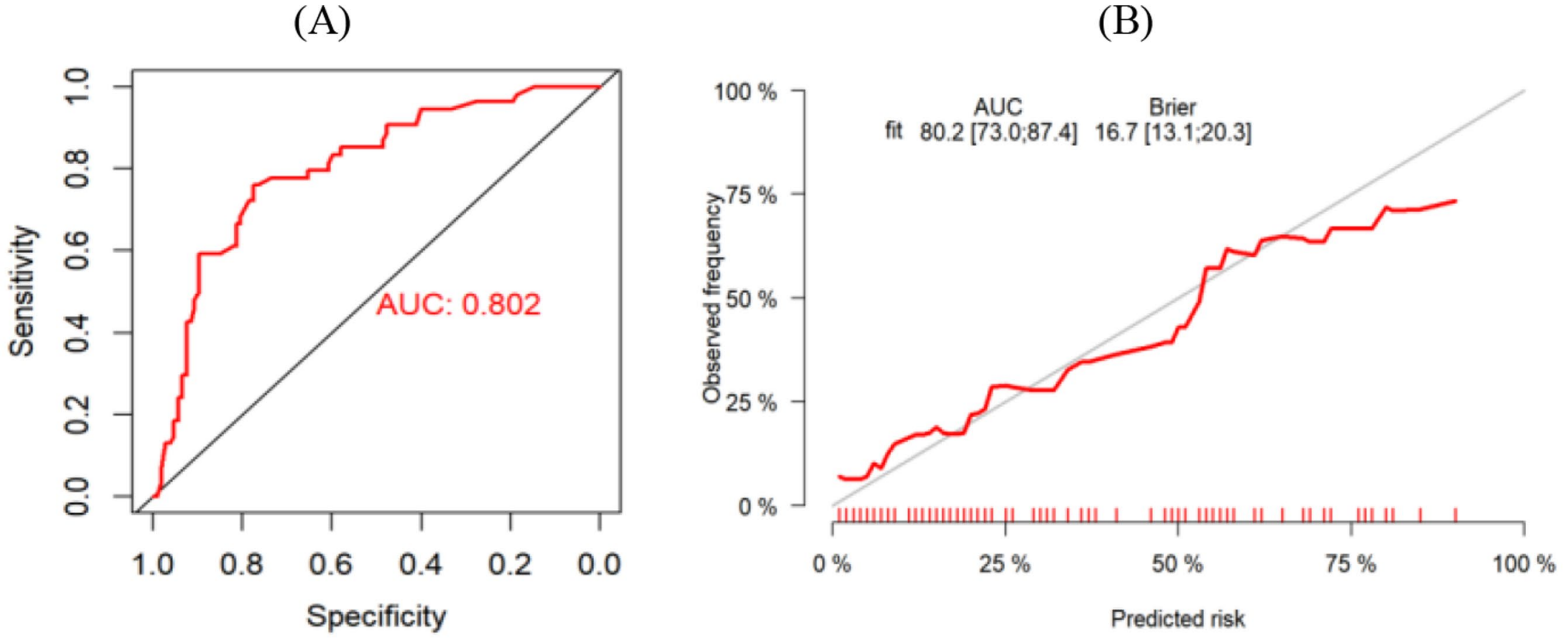
**(A)** ROC curve for risk predicton model for validation group. **(B)** Calibration curves of the validation group.

**Figure 7:**
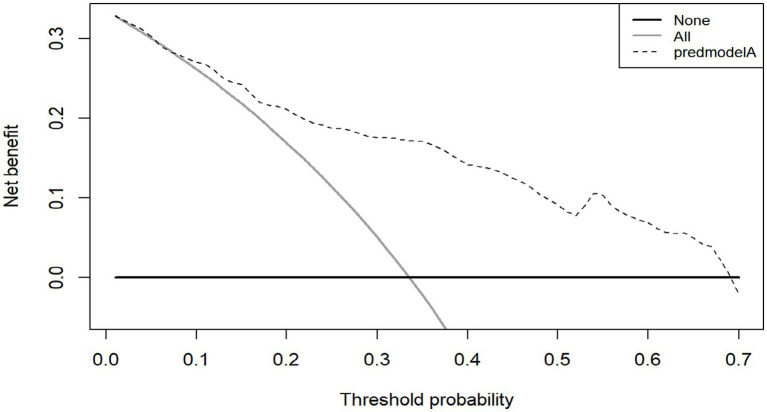
DCA of the validation group.

The AUC value of the validation group was 0.802 (95% CI, 0.730–0.874) (Figure 6), indicating that the model still had good discrimination in the external validation. Brier score was 0.167 (95% CI, 0.131–0.203); The calibration chart showed that the calibration curve of the model was close to that of the ideal model, and the probability of CF predicted by the model was in good agreement with the actual probability of CF in elderly patients with CRC (Figure 6). The H-L test results were: χ^2^ value = 9.100, *p* = 0.43, indicating that there was no statistically significant difference between the predicted risk and the actual risk of the model. The decision curve results are shown in Figure 7, where the use of the model provides a net benefit to clinical practice within a certain range of threshold probabilities, emphasizing the good clinical applicability of the model.

### 3.6 Drawing of the nomogram

**Figure 8:**
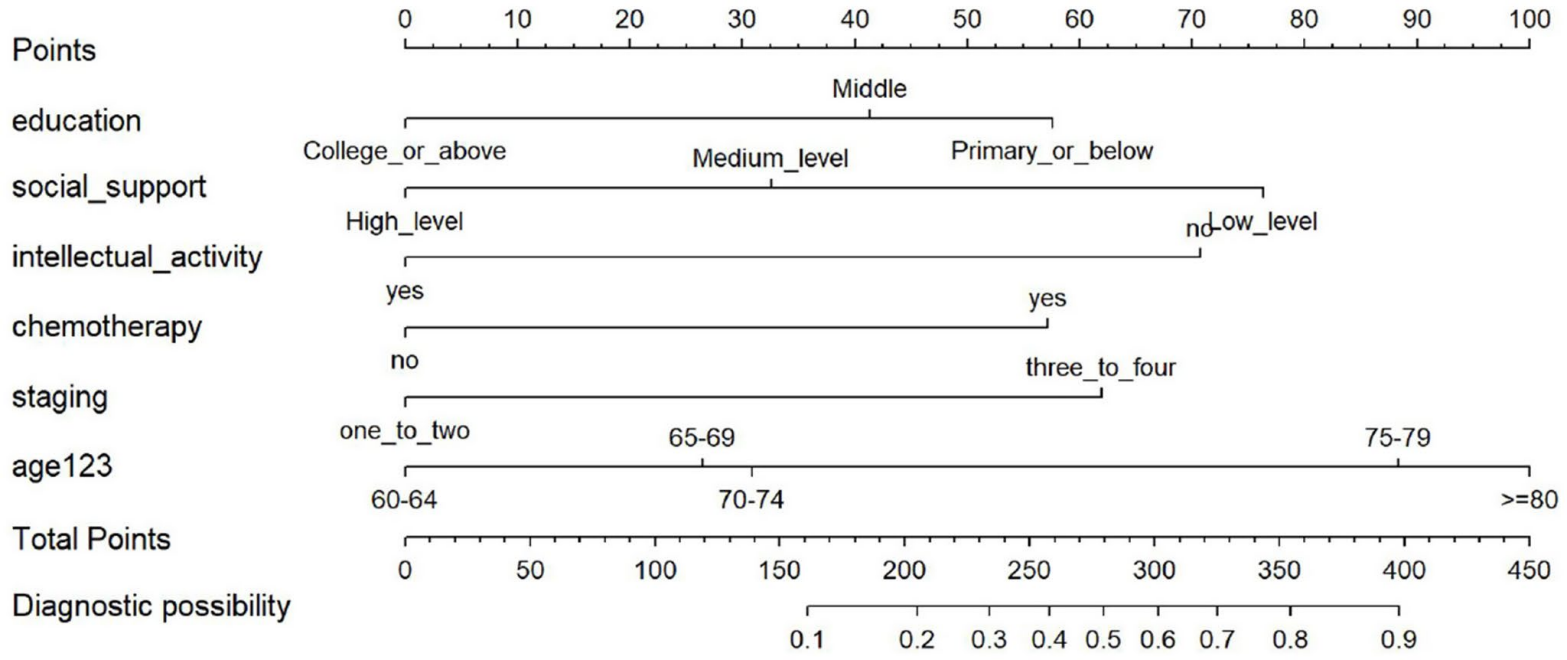
Nomogram for predicting the risk of CF.

In this study, R 4.4.3 software was used to establish a nomogram model that predicted the occurrence of CF in elderly patients with CRC, and the model was visualized. In this nomogram, the corresponding scores of each factor can be calculated according to the values of different predictors provided by the subjects. According to this principle, the risk scores of all predictors can be calculated in turn, and the total score after summing is the corresponding probability of CF, as shown in Figure 8.

### 3.7 Subgroup analysis

The World Health Organization (WHO) defines individuals aged ≥65 years as older adults in accordance with international standards. This study initially adopted the criterion of ≥60 years, primarily based on China’s national conditions and the fact that multiple national policies use this age as a threshold. To enhance the international comparability of the study, a subgroup analysis was conducted on patients aged ≥65 years. Results showed that the prediction model constructed in this study also exhibited good predictive performance in this subgroup (AUC = 0.818; H-L test: χ^2^ = 9.736, *p* = 0.284), indicating that the model has robust predictive efficacy for the elderly population as defined internationally.

Further subgroup validation was conducted for advanced-stage patients (stage III–IV) and patients with a history of chemotherapy, respectively. Results showed that for the advanced-stage patient subgroup, the AUC was 0.769, with a H-L test result of χ^2^ = 4.913 and *p* = 0.767. For the subgroup of patients with a history of chemotherapy, the AUC was 0.821, with an H-L test result of χ^2^ = 10.643 and *p* = 0.223. These findings indicate that the model still exhibits acceptable discriminative ability and calibration in advanced-stage patients and those who have received chemotherapy, with robust predictive efficacy.

A comparison between patients with stage III and stage IV tumors revealed no statistically significant difference in the risk of CF occurrence (OR = 0.528, 95% CI: 0.266–1.050, *p* = 0.069 > 0.05). Therefore, considering the trade-off between sample size and clinical heterogeneity, this study adopted a dichotomous staging strategy (stage I–II vs. stage III–IV) during model construction.

In addition, this study attempted to construct a simplified model that included only the 4 most impactful variables from the multivariate regression (age, staging, intellectual activity, and social support). For this simplified model, the AUC was 0.802 (95% CI, 0.756–0.848), and the H-L test yielded χ^2^ = 18.492 and *p* = 0.018. These results indicate poor calibration and a significant decline in model performance. Based on the trade-off between discriminative ability and multidimensional intervention value, we ultimately retained the six-variable model as the primary predictive model.

## 4 Discussion

CRC is a common malignancy in the elderly, requiring patients to have good cognitive, physical, and mental functions during diagnosis and treatment. However, the hidden nature of CF often results in delayed clinical assessment and missed early intervention opportunities.

The primary objective of our study was to develop an initial CF risk screening tool for the overall elderly CRC population. Risk management for cognitive frailty in this population is a continuous and dynamic process. If at-risk individuals are identified at the time of initial diagnosis—regardless of their disease stage—early and proactive intervention can be implemented, which may prevent or delay the development of severe CF. Additionally, the general screening model provides clinicians with a practical first step to rapidly stratify patients, thereby enabling more intensive and resource-intensive assessments for those identified as high-risk.

This study found that age ≥75 years is an independent risk factor for CF. This conclusion is consistent with previous studies (Michaud Maturana et al., 2021; Wen et al., 2021). As one ages, the body’s physiological reserves decline, leading to a disruption of homeostasis or a state of frailty. The related changes are manifested as a decline in muscle density, strength and mass, accelerated atrophy of key brain structures such as hippocampus and cerebral cortex, which leads to the dysfunction of susceptible neurons in specific brain systems. And the decline of brain structure and functional reserve, which is manifested as heterogeneous brain aging or CF (Gajdosova et al., 2023), increasing the risk of CF. Therefore, it is suggested that for patients aged 75 and above, special attention should be paid and early screening should be conducted. Based on the patient’s age and physical condition, an adaptive training program should be designed. For patients who are older and have relatively weaker physical functions, gentler exercise methods can be adopted, such as simple memory games and listening to stories while recalling details, to avoid overexertion.

Among 367 patients included in the modeling group of this study, 283 patients had a chemotherapy history, and 116 of them had CF, with an incidence of 31.61%. By LASSO regression, univariate and multivariate analysis, chemotherapy history was an independent risk factor for CF of patients (*p* = 0.002, β = 1.062). Patients with chemotherapy history were 2.892 times more likely to develop CF than those without chemotherapy (OR = 2.892, 95% CI: 1.463–5.715). The possible reason is that chemotherapeutic drugs can impair the mitochondrial structure and bioenergy of nerve cells, increase nitro-oxidative stress, and exert negative effects on a series of processes such as mitochondrial transport, fission, and fusion, thereby adversely affecting brain cells and metabolic functions (Doyle and Salvemini, 2021). In addition, chemotherapeutic drugs may increase the permeability of the blood–brain barrier by promoting the overexpression of inflammatory factors such as interleukin-6 (IL-6) and tumor necrosis factor-α (TNF-α). This allows inflammatory factors to rapidly enter the central nervous system (CNS), leading to an inflammatory response (Kesler et al., 2013). It further activates microglia to release more neurotoxic inflammatory mediators, including chemokines, prostaglandins, and nitric oxide. These mediators impair neuronal cells, subsequently inducing structural damage to the brain, and may ultimately contribute to varying degrees of impairment in learning and memory abilities, thereby leading to the development of cognitive frailty (Chae et al., 2016; Cheung et al., 2015). Therefore, prior to chemotherapy, medical staff can collaborate with specialists to assess the suitability of drugs that alleviate chemotherapy-related cognitive impairment for patients. During and after chemotherapy, specialized cognitive training programs should be developed for patients. Meanwhile, medical staff need to closely monitor patients’ responses during chemotherapy and timely adjust the training intensity based on patients’ tolerance levels.

Tumor stage is commonly used to evaluate the degree of malignancy of patients. The results of this study showed that the risk of CF in patients with tumor stage III–IV was 3.160 times that of patients with tumor stage I–II, that is, the higher the tumor stage, the greater the possibility of CF. Several studies have shown that tumor stage can affect cognitive status and frailty in patients (Li et al., 2023; Pereira et al., 2023). This might be due to the fact that advanced tumors (stage III to IV) have a large load and are highly invasive. Their active biological behavior is prone to causing physical weakness and CF (Li et al., 2023). Meanwhile, in advanced patients, the secretion of inflammatory factors (such as IL-6 and TNF-α) increases, which damages synaptic function and neural plasticity, and affects cognition (Guo and Lau, 2022). In addition, the deterioration of nutritional status is often accompanied by advanced stage patients, which may lead to impaired nerve conduction, changes in brain structure and reduced neuronal activity, and eventually show a decline in cognitive function. In addition, malnutrition can also aggravate the chronic inflammatory state and further promote neurodegenerative changes (Wang et al., 2024), thus accelerating the development of CF.

Furthermore, this study has confirmed that a lack of intellectual activities significantly increases the risk of CF, which is consistent with previous research (Song et al., 2022; Zhou et al., 2020; Feng et al., 2017). The reason may be that intellectual activity can make the brain more active and interfere with relevant pathological changes, thus reducing the occurrence of CF (Li et al., 2022). In addition, intellectual activities can protect and enhance cognitive functions by increasing cognitive stimulation of the brain, strengthening neuronal connections and plasticity (Verkhratsky and Zorec, 2024). For example, Fang and Shen (2017) found in their investigation on the intellectual activities and cognitive status of the elderly in the community that the more times they participated in intellectual activities, the greater the effect on improving their cognitive function, among which playing Musical Instruments and playing mahjong had the greatest effect on improving their cognitive function. Therefore, medical staff should help patients and their family members recognize the necessity of engaging in intellectual activities. They should encourage patients to participate in intellectual activities such as reading books and newspapers, practicing calligraphy, playing musical instruments, photography, painting, playing chess, and playing mahjong. On the premise that their physical condition permits, patients should be encouraged to expose themselves to more new things to promote brain activity, slow down the decline in cognitive ability, and thereby reduce the incidence of cognitive frailty to a certain extent.

The results of this study showed that the risk of CF in patients with low level of social support was 4.121 times that of high level of social support (OR = 4.121, 95% CI: 1.875–9.058). The degree of social support was an independent risk factor for CF of patients (*p* < 0.001). Social support comes from material or spiritual help and support obtained from others in social life. For elderly patients with CRC, the lack of spiritual support from family and friends can easily cause them to experience negative emotions such as loneliness, which can affect their cognitive functions and accelerate the process of CF. Previous studies have shown (Ning et al., 2021) that a high level of social support is beneficial to the development of physical and mental health of patients and can help patients build confidence in their disease. Therefore, medical staff should pay attention to and promptly assess patients’ social support levels, and understand their needs and deficiencies in terms of social support. They should encourage family members to spend more time accompanying patients in daily life to enhance emotional communication. Meanwhile, medical staff should help patients establish connections with community resources and coordinate various social support services.

The results of this study showed that education level of primary school or below was a risk factor for CF, indicating that there was a negative correlation between education level and CF risk, which was similar to the findings of previous studies (Li et al., 2024; Chen, 2020). Chen (2020) conducted a cross-sectional analysis on 245 elderly individuals aged 60 years and above, and Ruan et al. (2020) conducted a survey on elderly people in Shanghai communities. Both studies found that the higher the level of education, the lower the incidence of CF (or potential reversible CF). This might be because a higher level of education enhances brain plasticity, improves logical thinking, reasoning abilities, and the activity of brain cells, thereby increasing the density of brain synapses and neurons, and delaying brain aging (Li et al., 2018). On the other hand, studies by Wei Y et al. have found that educational level is a protective factor against physical frailty in older adults. Individuals with higher educational levels exhibit a higher level of self-health management, are more capable of taking the initiative to learn and acquire disease-related knowledge, and can adopt effective measures to prevent the occurrence of cognitive frailty (Wei et al., 2018). Therefore, these findings indicate that medical personnel should pay more attention to patients with lower educational levels, conduct timely screening, and adjust communication methods and content in a targeted manner according to the educational levels of different patients. Additionally, they should provide appropriate educational materials and interventions to delay the progression of cognitive frailty in these patients.

The Bootstrap 1,000 resampling method was used for internal validation, and the elderly patients with CRC from three Second-Class Grade A hospital in Qingdao were selected for external validation of the model. The predictive performance of the model was evaluated from three aspects: discriminative ability, calibration, and clinical validity. The AUC value in internal validation was 0.819 (95% CI, 0.775–0.863), and that in external validation was 0.802 (95% CI, 0.730–0.874), indicating that the model has a strong ability to discriminate whether cognitive frailty occurs in elderly patients with CRC; H-L goodness of fit test, calibration curve and Brier score were used to evaluate the calibration degree. The results of internal and external verification indicated that the prediction accuracy of the model was good and the risk predicted by the model was accurate. In addition to the above indicators, this study also analyzed the clinical effectiveness of the model through the decision curve, and the results showed that the model had good benefits in clinical application (Figure 5). To summarize, all performance metrics of this model in internal validation meet the standards. It can be used for the accurate prediction of the risk of developing CF in elderly patients with CRC, providing scientific and reliable tool support for clinical staff in their risk decision-making.

Based on the formula, the present study output the model in the form of a nomogram. It visualized the interactions between the effect sizes of various predictors of cognitive frailty, reduced the computational complexity of the model, and made the risk assessment of cognitive frailty in elderly patients with CRC simpler, more convenient, and easier to implement. Built on the formula-based model, the nomogram prediction model integrates risk factors with confirmed statistical significance to construct a graphical model, which enables quick retrieval of the risk of cognitive frailty occurrence. Based on the nomogram model, healthcare providers can intuitively predict the risk of CF and calculate its specific predicted probability according to the levels of various influencing factors in elderly patients with CRC. For high-risk patients, healthcare providers should take measures, implement personalized prevention strategies targeting the risk factors, enhance monitoring during hospitalization, and conduct dynamic assessments based on specific conditions to prevent or reverse the occurrence of cognitive frailty. The predictors involved in model construction in this study include age, history of chemotherapy, tumor stage, engagement in intellectual activities, social support, and educational level. These predictors cover research indicators related to both CF and CRC, and the scientific validity of the model has been further verified. Moreover, the above variables can all be identified and verified during admission assessment, which enhances the practicality of the results and allows its application in routine clinical settings.

The findings of this study suggest that advanced age, history of chemotherapy, tumor stage III–IV, lack of intellectual activities, low level of social support, and educational level of primary school or lower are risk factors for cognitive frailty in elderly patients with CRC. Risk management of cognitive frailty in this population requires close collaboration among multiple disciplines. As the primary healthcare providers with the closest contact with patients, nursing staff play a crucial role in the prevention of cognitive frailty. The model constructed in this study can serve as an important supplementary tool for assessing cognitive frailty in this population, thereby assisting medical and nursing staff in early identification and prediction of high-risk groups for cognitive frailty. Based on the various predictors in the model, medical and nursing staff should heighten vigilance when detecting any one of these high-risk factors in patients and adopt multiple measures to implement targeted interventions for high-risk patients. Therefore, it is necessary to enhance the theoretical knowledge and skills of specialized nursing staff related to cognitive frailty. In conjunction with the application of the prediction model, this will enable effective and timely monitoring of the developmental dynamics of cognitive frailty in elderly patients with CRC. Furthermore, elderly patients often have limited educational levels and ability to access healthcare-related information, which frequently leads to a lack of relevant knowledge. Thus, strengthening patient health education is critical. Through effective health education, patients can fully understand knowledge related to cognitive frailty, heighten their attention and awareness of the condition, and actively engage in daily activities and intellectual exercises. Accordingly, medical personnel can provide targeted health education to patients based on their individual conditions and in combination with the risk factors involved in the model of this study. This will help patients correctly understand and face cognitive frailty as well as its adverse effects on themselves, and actively cooperate with treatment and nursing work.

Limitations of the present study: Firstly, although external validation was conducted, all samples were collected from the same region, which limits the generalizability of the conclusions. Secondly, there are certain limitations in the candidate predictive variables of the model: factors such as cognitive-impacting medications, comorbidity information, specific chemotherapy purposes, and molecular markers were not included. Future studies need to incorporate these variables, which can further improve the predictive accuracy of the model and provide deeper insights into the biological underpinnings of CF in this population. On the other hand, in the fast-paced and busy clinical practice settings, the manual calculation method relied on by the nomogram often fails to achieve high efficiency. In the future, the model can be transformed into technological formats such as online calculators and mobile applications (APPs). These tools can generate the probability of outcome occurrence by inputting specific clinical information, which is convenient, efficient, and conducive to personalization, thereby meeting the needs of clinical workers for model application. Furthermore, due to time and human resource constraints, this study had a limited sample size, with a small number of patients with stage III and IV cancer included. The next step of research can enroll more patients with different cancer stages to explore in depth the association between cancer stage and the occurrence of CF. Oncology societies recommend the use of geriatric assessment tools such as G8 or IADL for elderly cancer patients, these tools were not used in this study. It is suggested that subsequent studies supplement these tools to enhance comprehensiveness. Finally, this study was entirely based on a cross-sectional study design, which can only analyze the association between potential factors and CF in elderly patients with CRC, but cannot directly evaluate the model’s ability to predict long-term outcomes and intervention effects. Future research may benefit from prospective longitudinal study designs to better understand the dynamic interactions between these variables over time. Given this, potential future research directions may include: conducting further multicenter, large-sample studies to improve the comprehensiveness of variable collection and the representativeness of sampling; considering comparisons of multiple modeling approaches such as machine learning to screen for the optimal model; and providing a more accurate predictive tool.

## 5 Conclusion

Against the backdrop of unclear risk factors and ambiguous status of CF occurrence in elderly patients with CRC, this study used methods including Lasso regression, univariate analysis, and multivariate Logistic regression to screen for the risk factors most relevant to CF. It further constructed a risk prediction model to conduct quantitative assessment of the risk of CF occurrence in this population. Furthermore, multicenter internal and external validations were conducted, and both confirmed that the model exhibited good discriminative ability and accuracy. This demonstrates that the prediction model has reliable performance, which is conducive to healthcare providers’ early identification of high-risk groups and implementation of targeted intervention measures.

## Data Availability

The original contributions presented in the study are included in the article/supplementary material, further inquiries can be directed to the corresponding authors.
