# Correction: Construction and validation of a cognitive frailty risk prediction model for elderly patients with colorectal cancer

**DOI:** 10.3389/fnagi.2025.1763134

**Published:** 2026-01-08

**Authors:** Yu Wang, Li Wang, Yunhong Du, Xiaoye Ma, Lili Sun, Xiujie Zhang, Wenli Rong, Jianwei Li, Yao Shi, Wei Liu, Danqi Xie, Lili Peng, Ouying Chen

**Affiliations:** 1 School of Nursing, Hunan University of Chinese Medicine, Changsha, China; 2 Nursing Department, Qingdao Traditional Chinese Medicine Hospital (Qingdao Hiser Hospital Affiliated of Qingdao University), Qingdao, China; 3 Nursing Department, The First Affiliated Hospital of Dalian Medical University, Dalian, China; 4 Cardiovascular Surgery Intensive Care Unit, The First Affiliated Hospital of Naval Medical University, Shanghai, China; 5 The First Clinical Medical College, Shandong University of Traditional Chinese Medicine, Jinan, China

**Keywords:** elderly patients, colorectal cancer, cognitive frailty, risk prediction model, nomogram

In the published article, there was a mistake in Figure 8 as published.

The corrected Figure 8 appears below.

**Figure 8:**
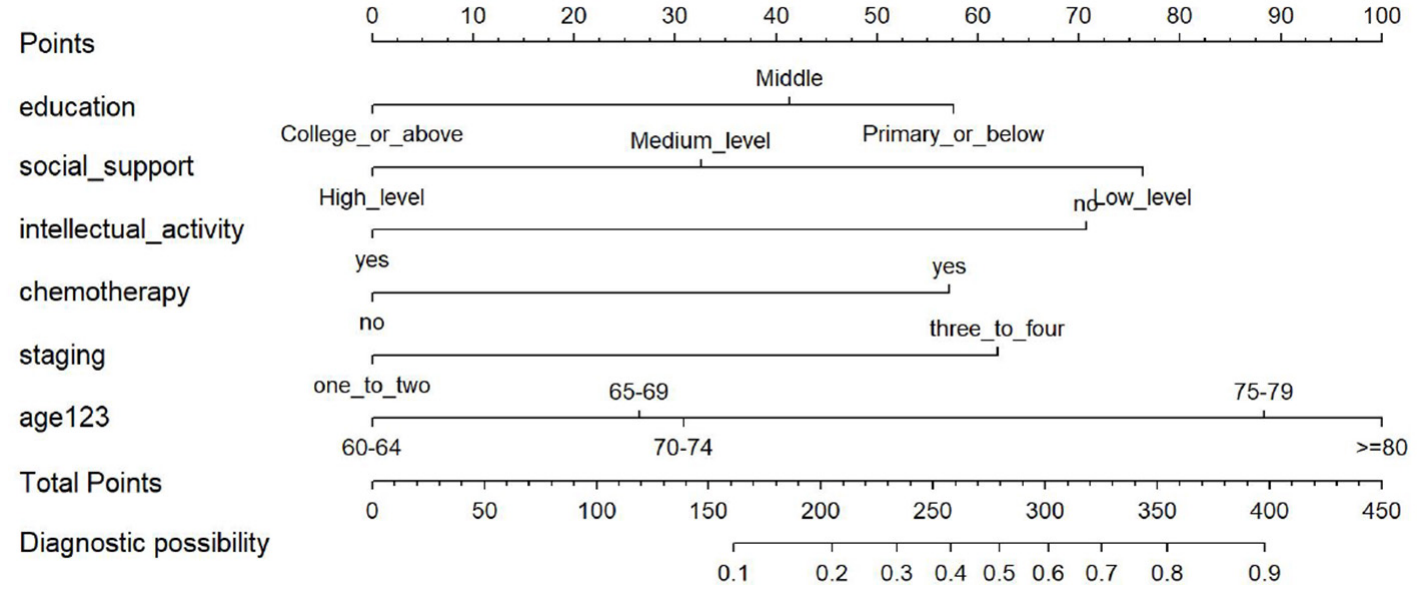
Nomogram for predicting the risk of CF.

The original version of this article has been updated.

